# Defining metabolic abnormalities in acute human traumatic brain injury with cerebral microdialysis and multimodality monitoring

**DOI:** 10.1371/journal.pone.0331310

**Published:** 2025-09-02

**Authors:** Michael S. Baker, Sara Venturini, Caroline Lindblad, Joshua M. Heihre, Ivan Timofeev, Mathew R. Guilfoyle, Peter J. Hutchinson, Keri L.H. Carpenter, Adel Helmy

**Affiliations:** 1 Division of Neurosurgery, Department of Clinical Neurosciences, University of Cambridge, Cambridge, United Kingdom; 2 Department of Medical Sciences, Section of Neurosurgery, Uppsala University, Uppsala, Sweden; 3 Department of Clinical Neuroscience, Karolinska Institutet, Stockholm, Sweden; Albany Medical College, UNITED STATES OF AMERICA

## Abstract

**Objective:**

We aimed to compare the prevalence and multimodal associations of mitochondrial dysfunction as defined by published cerebral-microdialysis-based criteria versus our novel multimodality-monitoring-based criteria in acute traumatic brain injury patients.

**Methods:**

We retrospectively analyzed neurocritical care monitoring data from 619 acute traumatic brain injury patients. Monitoring modalities included cerebral microdialysis, intracranial pressure, brain tissue oxygenation, cerebral perfusion pressure, and the pressure reactivity index. The cerebral-microdialysis-based criteria we compared combine an elevated lactate/pyruvate ratio (25 or 30) with raised concentrations of lactate (2.5 mM) or pyruvate (70 μM or 120 μM). Our multimodality-monitoring-based criteria comprise a consistent lactate/pyruvate ratio > 25 with intracranial pressure ≤ 20 mmHg, brain tissue oxygenation ≥ 15 mmHg, a pressure reactivity index ≤ 0.3, and cerebral glucose ≥ 1.0 mM.

**Results:**

Across 592 analyzable patients, a lactate/pyruvate ratio > 25 was common, with a median prevalence of 48.9% (41.5% with consistency) and a U-shaped, bimodal distribution. A lactate/pyruvate ratio > 25 was associated with lower glucose and higher glycerol, and when accompanied by high pyruvate (> 120 μM), this derangement was further distinguished by higher glutamate and cerebral perfusion pressure. Using multimodal criteria on a cohort of 268 patients, consistent mitochondrial dysfunction was identified in 25.7% to 41.0% of patients, often in the absence of other physiological derangements.

**Conclusions:**

Many acute traumatic brain injury patients constantly demonstrate neurometabolic derangements, among which clinical mitochondrial dysfunction is highly prevalent despite normal cerebral pressure, oxygenation, and perfusion. There is necessity for targeted, neurometabolic therapies in neurocritical care that address this abnormality.

## Introduction

Traumatic Brain Injury (TBI) is a condition with a complex underlying pathophysiology with several overlapping injurious mechanisms that contribute to secondary brain injury. A key component of these mechanisms is metabolic energy failure [1]. Cerebral microdialysis (CMD) is employed in neurocritical care to dynamically monitor brain extracellular fluid and can be used to identify deranged metabolism through markers such as a raised lactate/pyruvate ratio (LPR). The LPR is a direct indicator of cellular redox state and the ratio of NADH to NAD+ [2]. The clinical significance of these underlying processes is evident in that an increased LPR is associated with secondary injury as well as worse neurological [3–6] and brain tissue [7,8] outcomes. Thus, cerebral LPR in TBI patients has become a focus of clinical research and a therapeutic target [9–11].

In a systematic review parallel to the present study, we found that the literature uses an umbrella term of ‘metabolic crisis’ to describe differing metabolic states associated with a raised LPR [12]. Importantly, metabolic crisis can be subclassified into categories such as ischemia, when substrates such as glucose, pyruvate, or oxygen are insufficient to meet metabolic demands, and mitochondrial dysfunction, when substrates for aerobic metabolism are sufficient but inadequately utilized [10,12]. Furthermore, our systematic review demonstrated that variation exists in the criteria used for CMD-based clinical identification of mitochondrial dysfunction [12].

**Fig 1:**
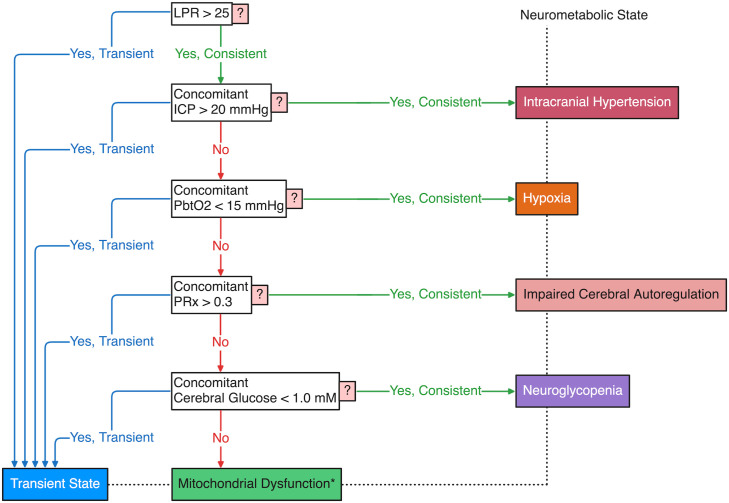
Hierarchical identification of neurometabolic states using multimodality monitoring. This protocol may be used for clinical identification of mitochondrial dysfunction if consistent LPR > 25 remains despite optimization of other multimodal parameters. We distinguish consistency from transience utilizing a novel algorithm described in Fig 2. *Mitochondrial dysfunction must be observed with consistency for identification. LPR, lactate/pyruvate ratio; ICP, intracranial pressure; PbtO2, brain tissue oxygenation; PRx, pressure reactivity index.

Different groups [13–17] have relied on CMD-based criteria of an LPR threshold of 25 or 30 combined with a lactate threshold of 2.5 mM or a pyruvate threshold of 70 μM or 120 μM for indication of mitochondrial dysfunction in patients (Table 1). In contrast, our group has proposed a CMD-inclusive, multimodality-monitoring-based (MMM-based) hierarchical framework for the clinical identification of mitochondrial dysfunction (Fig 1) [2,11]. Our MMM-based approach to identifying mitochondrial dysfunction requires a consistent LPR > 25 to trigger a tiered protocol of normalizing confounding conditions.

**Table 1:** Criteria suggested to indicate mitochondrial dysfunction.

MMM-based Criteria	References
Consistent LPR > 25 with ICP ≤ 20 mmHg, PbtO2 ≥ 15 mmHg, PRx ≤ 0.3, and cerebral glucose ≥ 1.0 mM (Fig 1)	Thelin et al. [2] and Khellaf et al. [11]
Alternative CMD-based Criteria	References
LPR > 25 with lactate > 2.5 mM	Sahuquillo et al. [13]
LPR > 25 with pyruvate > 70 μM	Gupta et al. [15]
LPR > 25 with pyruvate > 120 μM	Svedung Wettervik et al. [16] and Marini et al. [17]
LPR > 30 with pyruvate ≥ 70 μM	Nordström et al. [14]

MMM, multimodality monitoring; CMD, cerebral microdialysis; LPR, lactate/pyruvate ratio; ICP, intracranial pressure; PbtO2, brain tissue oxygenation; PRx, pressure reactivity index.

Our protocol first aims to achieve an intracranial pressure (ICP) ≤ 20 mmHg, then brain tissue oxygenation (PbtO2) ≥ 15 mmHg, next a pressure reactivity index (PRx) ≤ 0.3, and finally cerebral glucose ≥ 1.0 mM before defining mitochondrial dysfunction if the LPR is still consistently above 25 despite normalization of all other multimodal parameters. The PRx, a measure of cerebral autoregulation (CA), is calculated as the moving Pearson correlation coefficient between ICP and arterial blood pressure (ABP) over 5-minute buffers (30 consecutive 10-second windows) [18]. Hence, our protocol intends to systematically address potential etiologies for deranged LPR such that mitochondrial dysfunction can be identified with greater specificity: first resolving intracranial hypertension, then hypoxia, next impaired CA, and lastly neuroglycopenia. This tiered approach has clear implications for the potential therapeutic interventions available, with a view to incorporating CMD data into clinical management protocols.

Using data from a cohort of 619 TBI patients, the present retrospective analysis compares the prevalence of mitochondrial dysfunction in brain tissue as defined by various CMD-based approaches proposed by other groups [13–17] and as identified by our MMM-based hierarchical approach [2,11] (Table 1). This comparison evaluates the unmet need for therapeutics targeting deranged neurometabolism in TBI patients. Additionally, we assess the multimodal associations of such deranged neurometabolism with a view to gaining pathophysiological insight and discerning potential corrective therapies. To be thorough, we also explore the effects of modifying our MMM-based protocol for identifying neurometabolic states (NMS) and mitochondrial dysfunction with the CMD thresholds proposed by other groups. Ultimately, we aim to better understand the breadth and prevalence of metabolic abnormalities that occur following TBI as well as the potential to ameliorate such derangement.

## Materials and methods

### Study participants

Retrospective analysis was performed on a prospectively recruited cohort of 619 TBI patients admitted to the Addenbrooke’s neurocritical care unit, Cambridge, UK between December 5, 1997, and January 1, 2017. Verbal or written consent was obtained from the next of kin or legal representative at our tertiary center, with approval from both the Local Ethical Committee and the hospital Research and Development Department, as part of our head injury program. Consent was documented either physically or electronically. Participants were above 16 years of age with a median age of 37 (interquartile range of 24–52). As published previously [6], the cohort was 76.3% male and 23.7% female.

The patients were treated according to a standardized tiered therapy protocol first dictating sedation, paralysis, and mechanical ventilation to a target partial pressure of carbon dioxide of 4.5–5.0 kPa. A peripheral intra-arterial catheter was used for continuous ABP monitoring. Vasopressors were used to maintain a cerebral perfusion pressure (CPP) between 55 and 65 mmHg. An elevated ICP triggered a tiered approach with increasing intensity starting with osmotherapy, then external ventricular drainage, hypothermia, barbiturate coma, and, if still not resolved, decompressive craniectomy. Data analyses on the first 223 patients [3] recruited as well as the whole cohort of 619 patients [6] have been published previously.

### Monitoring

A triple lumen cranial access device (Technicam, Newton Abbot, UK) was placed in the non-dominant frontal lobe unless contraindicated and was used to introduce multimodal sensor tips to white matter. These invasive sensors included an ICP monitor (Codman, Raynham, MA, USA), a PbtO2 probe (Licox, Integra Neurosciences, Andover, UK), and a microdialysis catheter (CMA71, CMA/M Dialysis AB, Stockholm, Sweden; 100 kDa molecular weight cut-off, or CMA70, 20 kDa cutoff). The microdialysis catheter was perfused with standard crystalloid perfusate (CNS perfusion fluid, CMA/M Dialysis AB, Stockholm, Sweden) at flow rate of 0.3 μL/min.

ICP, ABP, and PbtO2 were recorded at 50–200 Hz using ICM+ software (ICM + , Cambridge Enterprise/University of Cambridge, UK). ICM + was used for bedside calculation of the PRx. Microdialysate was recovered hourly, and analyte concentrations were measured using bedside analyzers (CMA 600, ISCUS, or ISCUSflex; CMA/ M Dialysis AB, Stockholm, Sweden).

### Data processing

Data from CMD analyzers were imported using custom R scripts. An analyte’s value was discarded if the concentration was lower than the assay’s lower limit of detection, or if there was not enough microdialysate in a vial. The lower limits of the assays were: 0.1 mM for glucose, 0.1 mM for lactate, 10.0 μM for pyruvate, 1.0 μM for glutamate, and 10.0 μM for glycerol. The ICP, ABP, PRx, and PbtO2 data were averaged and aligned with the hourly CMD data while accounting for the 17-minute travel time of microdialysate from the intracranial catheter membrane to the external collection vial.

Winsorization of the data at the 0.00125 quantile was performed to remove bias from extreme outliers. This process changed data above the 99.875^th^ quantile and below the 0.125^th^ quantile to be equal to those respective quantile values without a reduction in the number of observations. For further data processing, the Python (v3.13.1, https://www.python.org/) package pandas [19] (v2.2.3) was used for dataframe manipulation.

**Fig 2:**
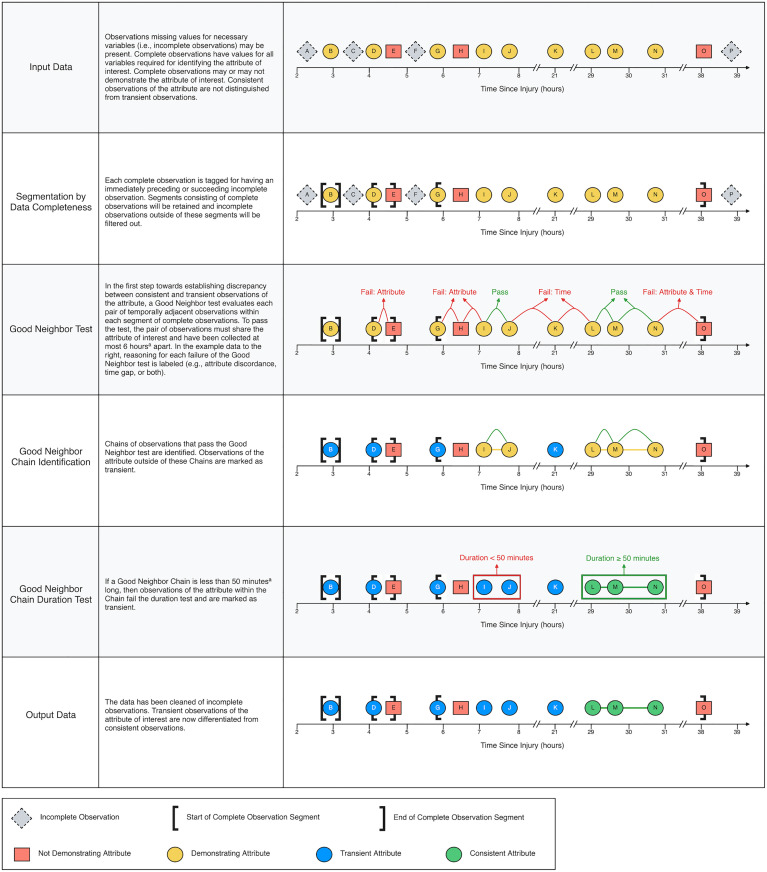
A two-parameter^a^ algorithm for differentiating consistent from transient attributes. The attribute may be unimodal (e.g., LPR > 25) or multimodal (e.g., LPR > 25 with ICP ≤ 20 mmHg, PbtO2 ≥ 15 mmHg, PRx ≤ 0.3, and cerebral glucose ≥ 1.0 mM, which our group proposes as suggesting mitochondrial dysfunction). ᵃTwo parameters: 1. maximum time gap (e.g., 6 hours) for Good Neighbor test. 2. minimum duration (e.g., 50 minutes) for Good Neighbor Chain duration test. LPR, lactate/pyruvate ratio; ICP, intracranial pressure; PbtO2, brain tissue oxygenation; PRx, pressure reactivity index.

Data were accessed for the present study on July 8, 2024. Any identifying information was anonymized prior to analysis. Prior to filtering, our dataset consisted of 56,307 multimodal observations across 619 patients. Throughout this manuscript, an ‘observation’ refers to a single time point for which any data, inclusive of CMD and physiological parameters, were collected. The first 2 hours of observations from each patient were excluded to mitigate the confounding impact of catheter insertion and probe flushing on microdialysis data. Then, we prepared our dataset for analyses which assess the intra-patient consistency of various CMD related and multimodal attributes through time (Fig 2). This additional quality control for ‘consistency’ was used to ensure that transient, potentially spurious values, did not interfere with our classification of neurometabolic abnormalities.

Our novel, two-parameter algorithm for differentiating consistent from transient attributes essentially relies on four steps: segmentation by data completeness, a ‘Good Neighbor’ (GN) test, ‘Good Neighbor Chain’ (GNC) identification, and a GNC duration test (Fig 2). The aggressiveness of the algorithm can be modulated by two parameters: the maximum time gap permitted between two observations of an attribute, which is used by the GN test, and the minimum acceptable duration of a GNC, which is used by the GNC duration test. Aiming to isolate observations of attributes grounded in real pathophysiology, and considering our typical hourly sampling resolution, we chose a maximum time gap of 6 hours for the GN test and a minimum duration of 50 minutes for the GNC duration test. Thus, for observations to be marked as consistently demonstrating an attribute: they must have been within a segment of multiple complete observations; must have shared the attribute with an immediately adjacent observation (i.e., preceding or succeeding, within the same segment) at most 6 hours apart (i.e., both observations passing the GN test); and must have been within a chain of such observations (i.e., a GNC) with a total duration of 50 minutes or longer (i.e., passing the GNC duration test).

For CMD-based analyses, observations were deemed incomplete and filtered out if they were missing data for the variables of lactate or pyruvate. These processing steps yielded a dataset of 51,729 observations across 592 patients, i.e., the complete CMD data cohort. For MMM-based analyses, observations were deemed incomplete and filtered out if they were missing data for the variables of ICP, PbtO2, PRx, cerebral glucose, or LPR (and lactate or pyruvate by association). These processing steps yielded a dataset of 17,046 observations across 268 patients, i.e., the complete MMM data cohort. The discrepancy in patient numbers between cohorts primarily arises from the lack of PbtO2 monitoring in earlier years.

### Statistical analysis

Statistical analysis was performed using Python (v3.13.1) and R (v4.4.2, https://www.r-project.org/). The Python package pandas [19] (v2.2.3) was used for data frame manipulation, the NumPy [20] package (v2.2.0) was used for numerical operations, and the Matplotlib [21] package (v3.10.0) was used for data visualization. The R packages readr, dplyr, and tidyr from the tidyverse [22] collection (v2.0.0) were used to read in, manipulate, and reshape the data, respectively.

Our statistical approach combined formal hypothesis testing for specific, predefined comparisons with descriptive statistics for overall cohort characterization. Formal comparisons, particularly those assessing differences between a parent cohort and a nested subgroup were analyzed using the robust bootstrap procedure detailed below. For other results, such as the prevalence of various NMS, we present descriptive statistics as point estimates representing our specific patient cohort.

To address the partially-paired data structure of our formal comparisons, where conventional tests like the standard t-test or Wilcoxon test are inappropriate, we employed a partially-paired bootstrap test. This computational method correctly preserves the dependency within the data. For each bootstrap iteration, we first resampled the indices of the paired patients (those present in both cohorts) and used that exact index vector to pull the corresponding observations from both cohorts, ensuring the pairing was maintained. The unpaired patients (present only in the parent cohort) were resampled separately. The reconstructed bootstrap-subgroup and bootstrap-parent-cohort were used to calculate a difference in central tendency, and this was repeated 100,000 times to generate a robust distribution of the effect size.

From this single bootstrap distribution, all key statistical outputs for formal comparisons were derived. The 95% confidence interval (CI) was taken from the 2.5th and 97.5th percentiles of the distribution. A two-tailed p-value was calculated by determining the proportion of simulated differences that fell on the opposite side of zero from the observed difference, then multiplying by two. For all comparisons, a difference was considered statistically significant if p < 0.05. No correction for multiple comparisons was performed because our study is primarily exploratory in nature, designed to compare a variety of predefined classification criteria and generate independent hypotheses.

### Presentation of results

All sample sizes (n) indicate number of patients. Values for LPR, lactate, pyruvate, glucose, glutamate, and glycerol were recorded from extracellular brain tissue using CMD, and so are cerebral values even if not explicitly stated. We use the term ‘consistency-negligent’ to refer to supersets including both transient and consistent occurrences. These groups are also referred to as the ‘Regardless of Consistency’ cohorts, in contrast to ‘With Consistency’ cohorts which have been filtered to contain only consistent occurrences. Furthermore, when discussing the results of our hierarchical classification protocol (Fig 1), we occasionally use the term ‘primary’ to emphasize that our classification rule assigns an observation only to the first NMS it meets in the sequence from intracranial hypertension through mitochondrial dysfunction. By this rule, mitochondrial dysfunction is only identified when it exists in isolation, after all other preceding NMS have been excluded.

## Results

### Prevalence of deranged CMD parameters

Our first set of analyses were CMD-based and focused on comparing mitochondrial dysfunction classification criteria without regard to the additional MMM-based criteria of our protocol. Alongside our LPR > 25 protocol trigger, we compared the four CMD-based classification criteria put forward by other groups in the peer-reviewed literature (Table 1).

**Fig 3:**
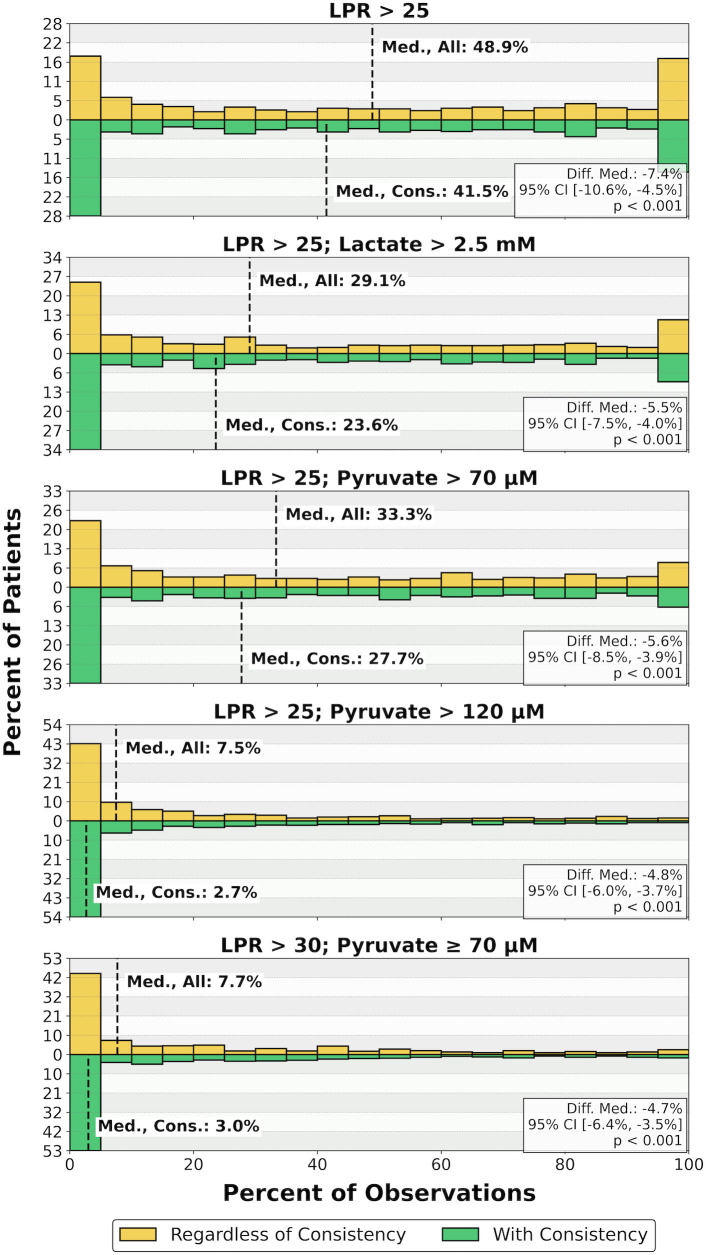
Distributions of patients’ percentage of observations meeting deranged CMD criteria regardless of consistency versus with consistency in the complete CMD data cohort (n = 592). For each set of criteria, the statistical comparison between the two distributions (difference in medians, 95% CI, and p-value) is displayed in a box at the bottom right. Difference in medians were calculated by subtracting that of the ‘With Consistency’ cohort from that of the ‘Regardless of Consistency’ (i.e., All) cohort. Except for the LPR > 25 criterion from our multimodality-monitoring-based classification protocol (Fig 1), used here without multimodality monitoring integration, all other deranged CMD criteria variants, regardless of consistency, were suggested by other groups to indicate mitochondrial dysfunction (Table 1). LPR, lactate/pyruvate ratio; Med., Median; Cons., Consistent; Diff. Med., Difference in Medians; 95% CI, 95% confidence interval; Regardless, Regardless of Consistency; CMD, cerebral microdialysis.

Each histogram in Fig 3 shows the distribution of patients’ percentage of observations meeting one of five deranged CMD criteria variants regardless of consistency (i.e., consistency-negligent, containing both transient and consistent occurrences) and with consistency in the complete CMD data cohort (n = 592). As for the consistency-negligent results, addition of a lactate > 2.5 mM criterion to the LPR > 25 criterion was accompanied by a decrease in median prevalence by 19.8% (48.9% to 29.1%). Addition of a pyruvate > 70 μM criterion to the LPR > 25 criterion was paired with less of a decrease in median prevalence by 15.6% (48.9% to 33.3%), and raising the pyruvate threshold from 70 μM to 120 μM yielded a further decrease in median prevalence by 25.8% (33.3% to 7.5%). The median prevalence of LPR > 30 with pyruvate ≥ 70 μM was 7.7%, and the raised LPR threshold of 30 corresponded with a 25.6% decreased median prevalence from that of LPR > 25 with pyruvate > 70 μM.

Overall, the distributions of prevalence of consistent CMD derangements were slightly more right skewed than the corresponding consistency-negligent prevalence distributions. Hence, the median prevalence for each deranged CMD criteria variant met with consistency was less than regardless of consistency: 41.5% for LPR > 25 (7.4% less), 23.6% for LPR > 25 with lactate > 2.5 mM (5.5% less), 27.7% for LPR > 25 with pyruvate > 70 μM (5.6% less), 2.7% for LPR > 25 with pyruvate > 120 μM (4.8% less), and 3.0% for LPR > 30 with pyruvate ≥ 70 μM (4.7% less). The two distributions (i.e., regardless of consistency and with consistency) for each deranged CMD criteria variant were significantly different from each other.

In line with the consistency-negligent results, the median percentage of observations consistently meeting deranged CMD criteria decreased with the addition of the lactate > 2.5 mM criterion (−17.9%) or the pyruvate > 70 μM criterion (−13.8%) to LPR > 25 alone and further decreased with a raised pyruvate threshold of 120 μM (−25.0%) compared to 70 μM. A rise in the LPR threshold from 25 to 30 with pyruvate > 70 μM also corresponded with a decrease in median prevalence (−24.7%).

Of note, U-shaped distributions can be seen for the LPR > 25 criterion variant and lesser so for the other deranged CMD criteria variants. Appropriately, Table 2 quantifies the prevalence of patients either never meeting or always meeting each deranged CMD criteria variant regardless of consistency and with consistency in the complete CMD data cohort (n = 592). As seen in Table 3, the percentage of patients showing CMD derangements at these extremes was even more pronounced in the complete MMM data cohort (n = 268), with no significant difference in the central tendency of these distributions versus the complete CMD data cohort (n = 592) as seen in Table 4.

**Table 2:** Patterns of patients meeting deranged CMD criteria^a^ regardless of consistency or with consistency^b^ in the complete CMD data cohort (n = 592).

Deranged CMD Criteria	Patients Never Meeting Criteria		Patients Always Meeting Criteria		Patients Both Meeting and Not Meeting Criteria	
	Regardless of Consistency	With Consistency	Regardless of Consistency	With Consistency	Regardless of Consistency	With Consistency
LPR > 25	56 (9.5%)	135 (22.8%)	66 (11.1%)	49 (8.3%)	470 (79.4%)	408 (68.9%)
LPR > 25; Lactate > 2.5 mM	86 (14.5%)	168 (28.4%)	30 (5.1%)	18 (3.0%)	476 (80.4%)	406 (68.6%)
LPR > 25; Pyruvate > 70 µM	87 (14.7%)	162 (27.4%)	24 (4.1%)	13 (2.2%)	481 (81.2%)	417 (70.4%)
LPR > 25; Pyruvate > 120 µM	174 (29.4%)	273 (46.1%)	5 (0.8%)	2 (0.3%)	413 (69.8%)	317 (53.5%)
LPR > 30; Pyruvate ≥ 70 µM	175 (29.6%)	274 (46.3%)	10 (1.7%)	6 (1.0%)	407 (68.8%)	312 (52.7%)

^a^The LPR > 25 criterion alone stems from our use of it as a trigger for our multimodality-monitoring-based classification protocol for identifying neurometabolic states and mitochondrial dysfunction (Fig 1), while other criteria were suggested by different groups to indicate mitochondrial dysfunction (Table 1).

^b^Investigation of consistency expands on the deranged CMD criteria suggested by other groups, which were consistency-negligent.

CMD, cerebral microdialysis; LPR, lactate/pyruvate ratio.

**Table 3:** Patterns of patients meeting deranged CMD criteria^a^ regardless of consistency or with consistency^b^ in the complete MMM data cohort (n = 268).

Deranged CMD Criteria	Patients Never Meeting Criteria		Patients Always Meeting Criteria		Patients Both Meeting and Not Meeting Criteria	
	Regardless of Consistency	With Consistency	Regardless of Consistency	With Consistency	Regardless of Consistency	With Consistency
LPR > 25	50 (18.7%)	74 (27.6%)	47 (17.5%)	36 (13.4%)	171 (63.8%)	158 (59.0%)
LPR > 25; Lactate > 2.5 mM	65 (24.3%)	94 (35.1%)	23 (8.6%)	17 (6.3%)	180 (67.2%)	157 (58.6%)
LPR > 25; Pyruvate > 70 µM	61 (22.8%)	89 (33.2%)	23 (8.6%)	16 (6.0%)	184 (68.7%)	163 (60.8%)
LPR > 25; Pyruvate > 120 µM	108 (40.3%)	136 (50.7%)	6 (2.2%)	3 (1.1%)	154 (57.5%)	129 (48.1%)
LPR > 30; Pyruvate ≥ 70 µM	103 (38.4%)	146 (54.5%)	11 (4.1%)	8 (3.0%)	154 (57.5%)	114 (42.5%)

^a^The LPR > 25 criterion alone stems from our use of it as a trigger for our MMM-based classification protocol for identifying neurometabolic states and mitochondrial dysfunction (Fig 1), while other criteria were suggested by different groups to indicate mitochondrial dysfunction (Table 1).

^b^Investigation of consistency expands on the deranged CMD criteria suggested by other groups, which were consistency-negligent.

CMD, cerebral microdialysis; MMM, multimodality monitoring; LPR, lactate/pyruvate ratio.

**Table 4:** Differences^a^ in central tendency of patients’ percentage of observations meeting deranged CMD criteria^b^ regardless of consistency or with consistency^c^ in the complete CMD data cohort (n = 592) versus the complete MMM data cohort (n = 268).

Deranged CMD Criteria	Regardless of Consistency	With Consistency
LPR > 25	−0.3% [−8.6%, 9.3%] (1.00)	−3.5% [−12.4%, 8.8%] (0.75)
LPR > 25; Lactate > 2.5 mM	−2.2% [−12.6%, 9.9%] (0.53)	−8.7% [−15.5%, 2.5%] (0.14)
LPR > 25; Pyruvate > 70 μM	1.2% [−10.4%, 11.5%] (0.99)	−5.0% [−14.7%, 7.7%] (0.46)
LPR > 25; Pyruvate > 120 μM	−2.4% [−4.9%, 0.8%] (0.11)	−2.7% [−3.8%, 1.2%] (0.31)
LPR > 30; Pyruvate ≥ 70 μM	−2.5% [−5.6%, 0.9%] (0.12)	−3.0% [−5.0%, 0.0%] (0.07)

^a^Differences are reported as: MMM cohort median ─ CMD cohort median [95% confidence interval] (p-value).

^b^The LPR > 25 criterion alone stems from our use of it as a trigger for our MMM-based classification protocol for identifying neurometabolic states and mitochondrial dysfunction (Fig 1), while other criteria were suggested by different groups to indicate mitochondrial dysfunction (Table 1).

^c^Investigation of consistency expands on the deranged CMD criteria suggested by other groups, which were consistency-negligent.

CMD, cerebral microdialysis; MMM, multimodality monitoring; LPR, lactate/pyruvate ratio.

### Multimodal context of consistently deranged CMD parameters

**Fig 4:**
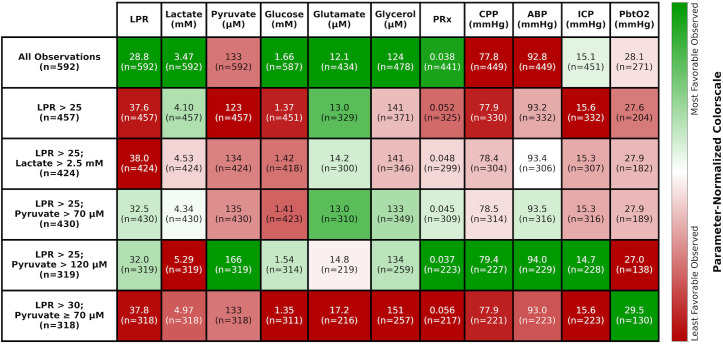
Means of patients’ means of parameters coinciding with consistently met deranged CMD criteria in the complete CMD data cohort (n = 592). Cell shading indicates favorability of the parameter value, from dark green being most favorable to dark red being least favorable observed in the column. The LPR > 25 criterion alone stems from our use of it as a trigger for our multimodality-monitoring-based classification protocol for identifying neurometabolic states and mitochondrial dysfunction (Fig 1), while other criteria were suggested by different groups to indicate mitochondrial dysfunction (Table 1). Results in this figure depend on the consistency of the CMD-based derangement, expanding on deranged CMD criteria suggested by other groups, which do not require consistency. CMD, cerebral microdialysis; LPR, lactate/pyruvate ratio; PRx, pressure reactivity index; CPP, cerebral perfusion pressure; ABP, arterial blood pressure; ICP, intracranial pressure; PbtO2, brain tissue oxygenation.

To understand the multimodal context for each of the five deranged CMD criteria variants being consistently met, we calculated patient-level means for each parameter of interest and then averaged these across all patients in the complete CMD data cohort (n = 592) so as to retain maximal data for each criteria-parameter combination (Fig 4). Differences in central tendency between select distributions of patients’ means of parameters coincident with consistent CMD derangements (e.g., LPR > 25 versus LPR > 25 with pyruvate > 70 μM) may be seen in Table 5.

**Table 5:** Differences^a^ in central tendency of patients’ means of parameters coinciding with differing consistently met deranged CMD criteria^b^ in the complete CMD data cohort (n = 592).

	LPR^c^	Lactate^c^ (mM)	Pyruvate^c^ (μM)	Glucose (mM)	Glutamate (μM)	Glycerol (μM)	PRx	CPP (mmHg)	ABP (mmHg)	ICP (mmHg)	PbtO2 (mmHg)
All Observations vs. LPR > 25	**8.8** **[7.6, 10.2]** (< 0.001*)	**0.63** **[0.54, 0.72]** (< 0.001*)	**−10** **[−14, −7]** (< 0.001*)	**−0.29** **[-0.36, -0.22]** (< 0.001*)	0.9 [-0.4, 2.2] (0.18)	**17** **[8, 25]** (< 0.001*)	**0.014** **[0.002, 0.027]** (0.03*)	0.1 [-0.4, 0.7] (0.68)	0.4 [-0.2, 1.0] (0.18)	**0.4** **[0.1, 0.8]** (0.02*)	−0.5 [-1.7, 0.7] (0.40)
All Observations vs. LPR > 25; Lactate > 2.5 mM	**9.2** **[7.8, 10.8]** (< 0.001*)	**1.06** **[0.96, 1.16]** (< 0.001*)	1 [−3, 5] (0.47)	**−0.24** **[-0.32, -0.16]** (< 0.001*)	**2.1** **[0.5, 3.7]** (0.01*)	**18** **[6, 28]** (0.002*)	0.010 [-0.005, 0.025] (0.18)	0.6 [-0.1, 1.2] (0.09)	0.6 [-0.2, 1.3] (0.12)	0.2 [-0.3, 0.7] (0.44)	−0.1 [-1.5, 1.3] (0.85)
All Observations vs. LPR > 25; Pyruvate > 70 μM	**3.7** **[2.4, 4.7]** (< 0.001*)	**0.86** **[0.76, 0.96]** (< 0.001*)	2 [−2, 5] (0.39)	**−0.25** **[-0.33, -0.17]** (< 0.001*)	0.9 [-0.5, 2.4] (0.19)	9 [−2, 20] (0.12)	0.006 [-0.008, 0.021] (0.39)	**0.7** **[0.0, 1.3]** (0.03*)	**0.7** **[0.0, 1.5]** (0.05*)	0.2 [-0.3, 0.6] (0.43)	−0.2 [-1.5, 1.2] (0.82)
All Observations vs. LPR > 25; Pyruvate > 120 μM	**3.2** **[1.8, 4.3]** (< 0.001*)	**1.82** **[1.69, 1.96]** (< 0.001*)	**33** **[29, 38]** (< 0.001*)	**−0.12** **[-0.22, -0.03]** (0.01*)	**2.7** **[0.6, 5.0]** (0.01*)	10 [−3, 23] (0.13)	−0.002 [-0.023, 0.019] (0.87)	**1.6** **[0.7, 2.6]** (< 0.001*)	**1.2** **[0.1, 2.2]** (0.03*)	−0.5 [-1.1, 0.2] (0.15)	−1.1 [-3.0, 0.8] (0.27)
All Observations vs. LPR > 30; Pyruvate ≥ 70 μM	**9.0** **[7.6, 10.1]** (< 0.001*)	**1.50** **[1.35, 1.66]** (< 0.001*)	0 [−4, 5] (0.89)	**−0.31** **[-0.41, -0.21]** (< 0.001*)	**5.1** **[2.5, 7.8]** (< 0.001*)	**28** **[13, 42]** (< 0.001*)	0.018 [-0.004, 0.040] (0.11)	0.1 [-0.8, 1.0] (0.88)	0.2 [-0.8, 1.3] (0.68)	0.4 [-0.2, 1.1] (0.22)	1.4 [-0.7, 3.6] (0.19)
LPR > 25 vs. LPR > 25; Lactate > 2.5 mM	0.4 [-0.3, 1.2] (0.26)	**0.43** **[0.39, 0.48]** (< 0.001*)	**12** **[10, 13]** (< 0.001*)	**0.06** **[0.01, 0.10]** (0.02*)	**1.2** **[0.5, 2.1]** (< 0.001*)	1 [−9, 9] (0.80)	−0.004 [-0.014, 0.006] (0.44)	**0.5** **[0.1, 0.8]** (0.02*)	0.2 [-0.2, 0.6] (0.42)	−0.2 [-0.5, 0.1] (0.11)	0.4 [-0.2, 1.0] (0.23)
LPR > 25 vs. LPR > 25; Pyruvate > 70 μM	**−5.1** **[-6.8, -3.6]** (< 0.001*)	**0.23** **[0.17, 0.29]** (< 0.001*)	**12** **[11, 13]** (< 0.001*)	**0.04** **[0.01, 0.07]** (0.006*)	0.1 [-0.7, 0.7] (0.85)	−8 [−18, 2] (0.11)	−0.008 [-0.017, 0.001] (0.08)	**0.6** **[0.2, 0.9]** (< 0.001*)	0.3 [-0.0, 0.7] (0.06)	**−0.3** **[-0.5, -0.0]** (0.03*)	0.4 [-0.1, 0.8] (0.11)
LPR > 25 vs. LPR > 25; Pyruvate > 120 μM	**−5.6** **[-7.6, -3.9]** (< 0.001*)	**1.19** **[1.08, 1.30]** (< 0.001*)	**43** **[41, 46]** (< 0.001*)	**0.17** **[0.10, 0.23]** (< 0.001*)	**1.8** **[0.3, 3.4]** (0.02*)	−6 [−19, 5] (0.30)	−0.016 [-0.036, 0.003] (0.11)	**1.5** **[0.7, 2.3]** (< 0.001*)	0.8 [-0.1, 1.6] (0.09)	**−0.9** **[-1.5, -0.3]** (0.002*)	−0.6 [-2.1, 0.9] (0.46)
LPR > 25; Pyruvate > 70 μM vs. LPR > 25; Pyruvate > 120 μM	**−0.5** **[-1.1, -0.0]** (0.03*)	**0.96** **[0.87, 1.05]** (< 0.001*)	**32** **[30, 33]** (< 0.001*)	**0.12** **[0.06, 0.18]** (< 0.001*)	**1.8** **[0.6, 3.1]** (0.004*)	1 [−8, 9] (0.77)	−0.008 [-0.026, 0.010] (0.38)	**0.9** **[0.2, 1.7]** (0.01*)	0.4 [-0.4, 1.3] (0.30)	**−0.7** **[-1.2, -0.1]** (0.02*)	−0.9 [-2.4, 0.5] (0.20)
LPR > 25; Pyruvate > 70 μM vs. LPR > 30; Pyruvate ≥ 70 μM	**5.2** **[4.9, 5.7]** (< 0.001*)	**0.64** **[0.53, 0.75]** (< 0.001*)	−1 [−4, 2] (0.41)	−0.06 [-0.13, 0.01] (0.09)	**4.2** **[2.4, 6.1]** (< 0.001*)	**18** **[9, 28]** (< 0.001*)	0.012 [-0.007, 0.030] (0.23)	−0.6 [-1.4, 0.1] (0.11)	−0.5 [-1.4, 0.3] (0.25)	0.2 [-0.3, 0.7] (0.34)	1.5 [-0.2, 3.3] (0.08)

*Significant p-value (p < 0.05).

^a^ Differences are reported as: Subgroup mean ─ Larger cohort mean [95% confidence interval] (p-value).

^b^ The LPR > 25 criterion alone stems from our use of it as a trigger for our multimodality-monitoring-based classification protocol for identifying neurometabolic states and mitochondrial dysfunction (Fig 1), while other criteria were suggested by different groups to indicate mitochondrial dysfunction (Table 1). Investigation of consistency expands on the deranged CMD criteria suggested by other groups, which were consistency-negligent.

^c^ For comparisons involving one of the filtering variables (e.g., comparing LPR after applying a filter of LPR > 25), the resulting p-value should be interpreted with caution. It serves as a measure of the filter’s impact and statistical reliability; a non-significant result would indicate that the filter, while applied, did not produce a statistically detectable shift in the group’s central tendency. The difference in means and its 95% CI are provided to quantify the magnitude of this effect.

CMD, cerebral microdialysis; LPR, lactate/pyruvate ratio; PRx, pressure reactivity index; CPP, cerebral perfusion pressure; ABP, arterial blood pressure; ICP, intracranial pressure; PbtO2, brain tissue oxygenation.

Compared to cerebral glucose across all observations (mean glucose 1.66 mM), glucose was significantly lower with any of the consistent CMD derangements of interest (range mean glucose 1.35 mM to 1.54 mM). Compared to glutamate across all observations (mean glutamate 12.1 μM), glutamate was significantly higher when concomitant with any of the consistent CMD derangements of interest (range mean glutamate 14.2 μM to 17.2 μM), excluding the LPR > 25 subset and the LPR > 25 with pyruvate > 70 μM subset (both with mean glutamate 13.0 μM). Compared to glycerol across all observations (mean glycerol 124 μM), glycerol was significantly higher when concomitant with any of the consistent CMD derangements of interest (range mean glycerol 141 μM to 151 μM), excluding the LPR > 25 with raised pyruvate (> 70 μM and > 120 μM) subsets.

PRx across all observations (mean PRx 0.038) was only significantly lower than that of the consistent LPR > 25 subset (mean PRx 0.052, p = 0.03). CPP across all observations (mean CPP 77.8 mmHg) was significantly lower than that of the subsets of observations with consistent LPR > 25 with pyruvate > 70 μM (mean CPP 78.5 mmHg, p = 0.03) and LPR > 25 with pyruvate > 120 μM (mean CPP 79.4 mmHg, p < 0.001). Relatedly, ABP across all observations (mean ABP 92.8 mmHg) was significantly lower than that of the subsets of observations with consistent LPR > 25 with pyruvate > 70 μM (mean ABP 93.5 mmHg, p = 0.05) and LPR > 25 with pyruvate > 120 μM (mean ABP 94.0 mmHg, p = 0.03). ICP across all observations (mean ICP 15.1 mmHg) was only significantly lower than that of the consistent LPR > 25 subset (mean ICP 15.6 mmHg, p = 0.02). There were no significant differences found between select subsets of interest (i.e., all observations or demonstrating select, consistent CMD derangement) for PbtO2.

### Prevalence of consistent neurometabolic states

**Fig 5:**
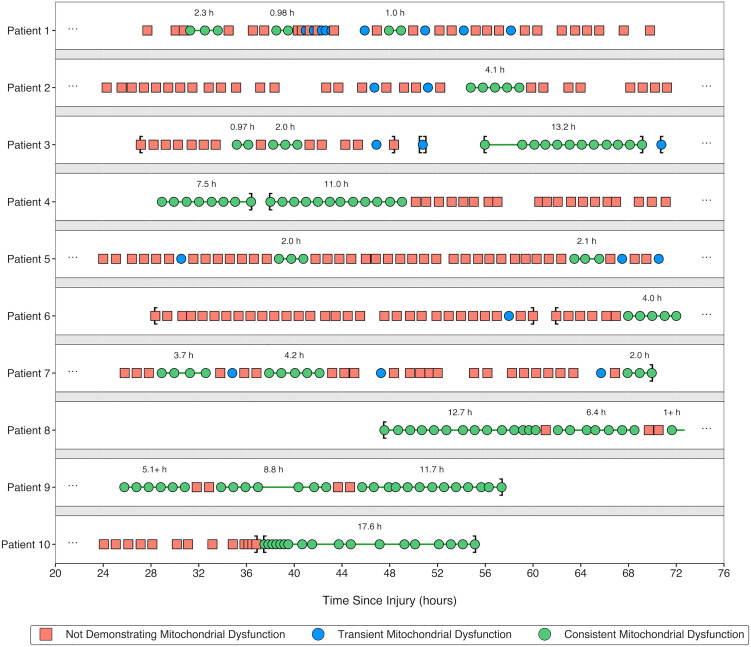
Select results from algorithmic differentiation of consistent from transient mitochondrial dysfunction identified using our hierarchical MMM-based classification protocol (with a trigger of LPR > 25) (Fig 1). MMM, multimodality monitoring; LPR, lactate/pyruvate ratio.

We then shifted our focus toward incorporating MMM as detailed in our protocol (Fig 1) and integrating the CMD-based criteria proposed by other groups into such a hierarchical framework for defining NMS, including mitochondrial dysfunction. Table 6 demonstrates that for deranged CMD criteria integrated into our MMM-based protocol, there was a significant difference in the percentage of observations identified as having isolated mitochondrial dysfunction regardless of consistency versus with consistency. Fig 5 visualizes the effect on select patient data series of our process for algorithmically differentiating consistent from transient attributes (Fig 2) when applied to mitochondrial dysfunction.

**Table 6:** Differences in central tendency of patients’ percentage of observations demonstrating hierarchically identified mitochondrial dysfunction regardless of consistency versus with consistency in the complete MMM data cohort (n = 268).

Deranged CMD Trigger^a^	Mean Percentages of Observations With Mitochondrial Dysfunction		Differences^b^ in Means
	Regardless of Consistency	With Consistency	
LPR > 25	13.8%	11.3%	−2.5% [−3.0%, −2.0%] (< 0.001*)
LPR > 25; Lactate > 2.5 mM	12.5%	10.1%	−2.4% [−2.9%, −1.9%] (< 0.001*)
LPR > 25; Pyruvate > 70 μM	13.1%	10.6%	−2.4% [−3.0%, −1.9%] (< 0.001*)
LPR > 25; Pyruvate > 120 μM	8.7%	6.8%	−1.8% [−2.3%, −1.4%] (< 0.001*)
LPR > 30; Pyruvate ≥ 70 μM	7.5%	6.1%	−1.4% [−1.9%, −1.1%] (< 0.001*)

*Significant p-value (p < 0.05)

^a^Our MMM-based classification protocol originally uses the trigger of LPR > 25 (Fig 1), but here we also investigate the impact of using alternative triggers as inspired by the deranged CMD criteria for mitochondrial dysfunction suggested by other groups (Table 1).

^b^Differences are reported as: ‘With Consistency’ mean─ consistency-negligent mean [95% confidence interval] (p-value)

MMM, multimodality monitoring; CMD, cerebral microdialysis; LPR, lactate/pyruvate ratio.

**Fig 6:**
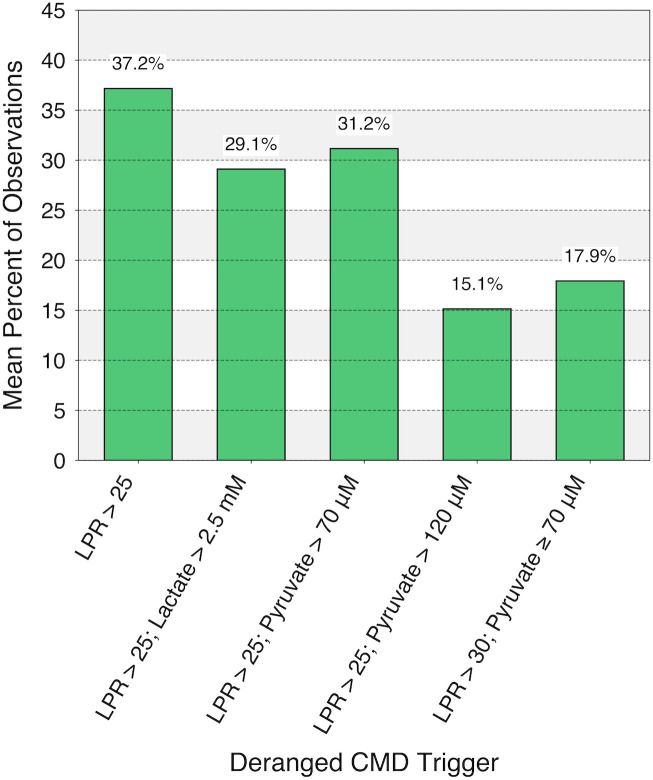
Means of patients’ percentage of observations demonstrating consistent neurometabolic states in the complete MMM data cohort (n = 268). Each bar displays the results corresponding to one of five variations in the CMD-based trigger for our hierarchical MMM-based classification protocol, originally using the trigger of LPR > 25 (Fig 1). MMM, multimodality monitoring; CMD, cerebral microdialysis; LPR, lactate/pyruvate ratio.

As seen in Table 7, the means of patients’ percentage of observations not meeting each of the deranged CMD triggers (i.e., the ‘No CMD Derangement’ column) necessary for classification into an NMS ranged from 50.7% to 79.2% depending on the CMD-based triggers used. The means of patients’ percentage of observations meeting deranged CMD criteria yet only being classified transiently into an NMS (i.e., the ‘Transient State’ column) ranged from 5.7% to 12.2%. Thus, the mean prevalence of consistent NMS being met (100% − ‘No CMD Derangement’ – ‘Transient State’) was highest using the LPR > 25 trigger (mean 37.2%) and lowest using the LPR > 25 with pyruvate > 120 μM trigger (mean 15.1%) (Fig 6). Across the different variations on our protocol, the mean prevalence of each primary, consistent NMS, as defined by our hierarchical classification (Fig 1), was then determined. The prevalence of intracranial hypertension as the primary derangement ranged from 2.8% to 8.9%, hypoxia from 1.5% to 4.1%, impaired CA from 1.6% to 3.0%, neuroglycopenia from 2.4% to 9.9%, and mitochondrial dysfunction as an isolated finding from 6.1% to 11.3%. Notably, the total percentage of patients demonstrating any instance of isolated, consistent mitochondrial dysfunction at all throughout their monitoring ranged from 25.7% to 41.0%.

**Table 7:** Prevalence of neurometabolic states identified by hierarchical classification in the complete MMM data cohort (n = 268).

Deranged CMD Trigger^a^	Mean Percentages of Observations Demonstrating							Patients Demonstrating Any Consistent Mitochondrial Dysfunction
	No CMD Derangement	CMD Derangement						
		Transient State	Consistent State^b^					
			Intracranial Hypertension	Hypoxia	Impaired CA	Neuroglycopenia	Mitochondrial Dysfunction	
LPR > 25	50.7%	12.2%	8.9%	4.1%	3.0%	9.9%	11.3%	110 (41.0%)
LPR > 25; Lactate > 2.5 mM	61.1%	9.8%	6.3%	3.1%	2.6%	7.0%	10.1%	101 (37.7%)
LPR > 25; Pyruvate > 70 µM	58.1%	10.7%	6.6%	3.1%	2.7%	8.2%	10.6%	106 (39.6%)
LPR > 25; Pyruvate > 120 µM	79.2%	5.7%	2.8%	1.5%	1.6%	2.4%	6.8%	78 (29.1%)
LPR > 30; Pyruvate ≥ 70 µM	75.8%	6.3%	4.1%	1.5%	1.8%	4.5%	6.1%	69 (25.7%)

^a^ Our MMM-based classification protocol originally uses the trigger of LPR > 25 (Fig 1), but here we also investigate the impact using alternative triggers as inspired by the deranged CMD criteria for mitochondrial dysfunction suggested by other groups (Table 1).

^b^ The ‘Consistent State’ columns represent mutually exclusive categories based on the hierarchical framework shown in Fig 1. An observation is assigned only to the first derangement met in the sequence from intracranial hypertension through mitochondrial dysfunction.

MMM, multimodality monitoring; CMD, cerebral microdialysis; LPR, lactate/pyruvate ratio; CA, cerebral autoregulation.

**Fig 7:**
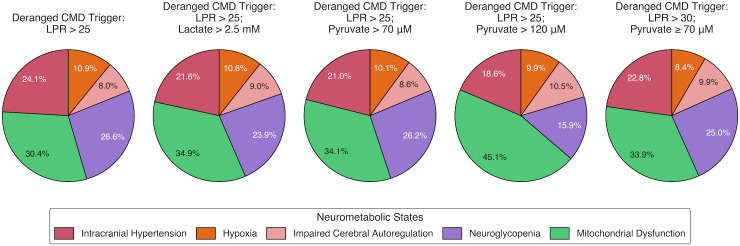
Relative distribution of consistent neurometabolic states identified by hierarchical classification in the complete MMM data cohort (n = 268). Each pie chart displays the 100%-normalized means of patients’ percentage of observations demonstrating a consistent neurometabolic state, corresponding to one of five variations in the CMD-based trigger for our MMM-based classification protocol that originally uses the trigger of LPR > 25 (Fig 1). Crucially, these neurometabolic states are mutually exclusive, as each observation is assigned only to the first derangement identified in the hierarchical protocol from intracranial hypertension through mitochondrial dysfunction. For example, observations with both high ICP and hypoxia are classified solely as intracranial hypertension. CMD, cerebral microdialysis; LPR, lactate/pyruvate ratio; MMM, multimodality monitoring.

Fig 7 displays 100%-normalized mean percentages of the five consistent NMS identified using each variant of our hierarchical MMM-based protocol (Table 7). Within these consistent NMS (Fig 1), mitochondrial dysfunction in isolation was the most represented for each of the five variants (range of 30.4% to 45.1%). Hierarchically identified (i.e., primary) intracranial hypertension (range of 18.6% to 24.1%) and neuroglycopenia (range of 15.9% to 26.6%) were the next most represented, with neuroglycopenia slightly more dominant than intracranial hypertension for all criteria variations except for the LPR > 25 with pyruvate > 120 μM variant. Primary hypoxia (range of 8.4% to 10.9%) and impaired CA (range of 8.0% to 10.5%) were the least represented, with hypoxia slightly more prominent than impaired CA for criteria variations excluding those using the trigger of consistent LPR > 25 with pyruvate > 120 μM or LPR > 30 with pyruvate ≥ 70 μM.

Although our original LPR > 25 trigger was associated with the highest absolute mean prevalence of isolated, consistent mitochondrial dysfunction (11.3%) (Table 7), it conferred the lowest relative (100%-normalized) mean prevalence of mitochondrial dysfunction when compared to all other primary, consistent NMS (30.4%) (Fig 7). Displaying similar discordance, despite the LPR > 25 with pyruvate > 120 μM variant’s lower absolute mean prevalence of consistent mitochondrial dysfunction (6.8%) than the LPR > 25 with pyruvate > 70 μM variant (10.6%), the raised pyruvate threshold was associated with the highest relative mean prevalence of consistent mitochondrial dysfunction (45.1%) out of all criteria variants.

## Discussion

While the LPR reflects a final common derangement of cellular redox state following a range of metabolic disturbances, a classification that identifies the cause of the LPR disturbance is required to appropriately enact clinical therapies. The data presented in the current study reflect the breadth and prevalence of the metabolic and related multimodal disturbances that are associated with elevated LPR with a view to incorporating microdialysis monitoring into clinical protocols.

We have demonstrated that select variants of criteria for identifying mitochondrial dysfunction (Table 1) are distinct from each other in prevalence of being met and in concomitant multimodal associations. This could have been anticipated from the differing aims of each set of criteria. Although the LPR > 25 with lactate > 2.5 mM variant specifies a certain level of anaerobic byproduct with a lactate threshold, it should be noted that lactate can also be used as an energy substrate in the traumatically injured brain, with some evidence that it is preferred by neurons [23]. Therefore, each criteria variant that we have studied requires a differing level of substrate provision prior to identifying mitochondrial dysfunction with an elevated LPR. Included in our classification protocol are the requirements of cerebral glucose ≥ 1.0 mM and PbtO2 ≥ 15 mmHg (Fig 1), while other groups have proposed thresholds for pyruvate or lactate provision.

### Identification of CMD-based derangements

Out of all CMD derangements investigated, LPR > 25, representing our protocol’s trigger, had the greatest prevalence. It was observed in about half of monitored observations by median regardless of consistency and exclusively with consistency (48.9% and 41.5%, respectively) in the complete CMD data cohort (n = 592) (Fig 3). The prevalence of consistent CMD derangements shows us how often our MMM protocol for isolating mitochondrial dysfunction may be triggered. Thus, there would be a large difference in how often our protocol would be enacted using differing CMD-based triggers, ranging from a median 41.5% of monitoring time with a trigger of consistent LPR > 25 to just 2.7% with a trigger of consistent LPR > 25 with pyruvate > 120 μM.

Remarkably, a U-shaped distribution was most prominent for the prevalence of LPR > 25 (Fig 3), owing to a substantial percentage of patients either never or always demonstrating the derangement (Tables 2 and 3). In a recent prospective study of 33 TBI patients, our group found a similar U-shaped distribution of LPR > 25 with approximately 4 patients (12.0%) at each extreme [11]. These findings suggest a biological difference in metabolic response to injury between cohorts at these two extremes; the etiology of this polarization has yet to be described. Importantly, the present study found no significant difference in the distributions of prevalence of CMD derangements between our complete CMD data cohort (n = 592) and our complete MMM data cohort (n = 268) (Table 4), suggesting that our findings may be generalizable between cohorts and a lack of bias in our more selective complete MMM data cohort of patients who received more comprehensive monitoring.

### Multimodal milieu of consistent CMD-based derangements

Notably, all variants of consistent CMD derangements that we explored had significantly lower levels of glucose than the population of all observations. This was coupled with a trend toward higher glutamate and glycerol—markers of excitotoxicity and cell damage—which reached statistical significance in most, but not all, of these conditions.

Consistent LPR > 25 with pyruvate > 120 μM had the highest concomitant mean energy substrate levels of glucose and pyruvate out of all CMD derangements explored, so concomitantly increased glutamate, a marker of excitotoxicity, is not intuitive. However, the increased glutamate may have been upregulating the astrocyte-neuron lactate shuttle, driving aerobic respiration and release of extracellular lactate from astrocytes [24,25]. This is corroborated by consistent LPR > 25 with pyruvate > 120 μM being associated with the lowest LPR and highest lactate levels out of all derangements explored.

Lastly, both of the consistent LPR > 25 with high pyruvate criteria (> 70 μM and > 120 μM) were associated with significantly higher CPP compared to the population of all observations. Particularly, the increased CPP was accompanied by significantly increased ABP, but no significant change in ICP. The upward shift in the CPP point estimate and confidence interval with the stricter pyruvate threshold suggests that CPP escalation could be used to prospectively manipulate brain extracellular pyruvate.

### MMM-identified neurometabolic states

Our protocol identifies five conditions in a stepwise fashion (Fig 1), which we refer to as neurometabolic states (NMS) owing to their neuropathophysiology coinciding with deranged metabolism (e.g., high LPR). These states range from intracranial hypertension to mitochondrial dysfunction, and the design of the hierarchical protocol was guided by several principles intended to reflect a pragmatic, clinically-oriented workflow. For instance, placing hypoxia before impaired autoregulation prioritizes a more readily addressable physiological goal, while the use of PRx over a static CPP target provides a more personalized assessment of cerebrovascular health when patient-specific optimal CPP (i.e., CPP-opt) cannot be determined from hourly data. Finally, we chose cerebral glucose over pyruvate as the marker for substrate availability to provide a direct indicator of upstream nutrient delivery while avoiding the endogeneity that would arise from using pyruvate in a protocol that relies on the LPR for its final classification. Our retrospective, statistical exploration of these NMS lends insight not only into the implications of our MMM-based protocol, but also the effect of modifying our protocol to utilize alternative deranged CMD triggers inspired by criteria proposed by other groups such as various lactate or pyruvate thresholds or a raised LPR threshold (Table 1) compared to the trigger of LPR > 25 alone as our protocol currently stands.

Retrospective application of our MMM-based classification protocol with our original trigger of consistent LPR > 25 exposed that almost half (41.0%) of patients demonstrated isolated, consistent mitochondrial dysfunction at some point during their monitoring (Table 7). We suspect that this percentage of patients is less than the 72.7% we have previously observed [11] in a prospective cohort (n = 33) due to our use of a more stringent algorithm for defining consistency in the present study (Fig 2). Nonetheless, this represents a large cohort of patients that may benefit from specialized treatment targeting deranged neurometabolism during their care, from manipulating multimodal parameters to pharmacological therapy such as succinate administration [9–11]. Even in integrating deranged CMD criteria suggested by other groups into our MMM-based protocol for identifying mitochondrial dysfunction, this cohort of neurometabolic concern still comprised over a fourth (25.7% at the least) of patients.

Regardless of the CMD-based trigger combined with our MMM-based protocol, mitochondrial dysfunction was the most common hierarchically identified NMS, followed by intracranial hypertension or neuroglycopenia, and the least common NMS were hypoxia and impaired CA (Fig 7). To reiterate the hierarchical nature of our protocol’s definition of NMS (Fig 1): the NMS of intracranial hypertension obfuscates any instances of concomitant hypoxia, impaired CA, neuroglycopenia, and mitochondrial dysfunction; likewise, the NMS of hypoxia obfuscates impaired CA, neuroglycopenia, and mitochondrial dysfunction, and so on. Nonetheless, our results suggest that consistent mitochondrial dysfunction can most often be isolated without coexistent derangements in multimodal parameters such as ICP, PbtO2, PRx, or glucose, further indicating that mitochondrial dysfunction deserves targeted treatment of its own.

### Limitations and future directions

Inherent in our study design was a level of overlap between comparison groups. For instance, observations meeting more stringent criteria such as LPR > 25 with pyruvate > 120 μM will also belong to the subset of observations meeting the criteria of LPR > 25 with pyruvate > 70 μM. Although our careful selection of statistical techniques to handle such partially-paired (i.e., nested) data ensured the validity of our results, this overlap present in our comparisons likely attenuated the differences between groups. Thus, our findings may be seen as conservative. Considering this nuance, we believe that our study has identified robust differences between these classification algorithms.

It may seem that our protocol’s hierarchical approach to identifying mitochondrial dysfunction does not fully capture the coexistence of multiple abnormalities or NMS. For example, true mitochondrial dysfunction at the cellular level may actually be occurring regardless of a simultaneously identified NMS such as intracranial hypertension. However, our protocol’s goal is to systematically and linearly address confounding conditions so that the indications of mitochondrial dysfunction (e.g., raised LPR) can be isolated as most likely stemming from metabolic crisis at the cellular level. Thus, the NMS of mitochondrial dysfunction that we identify using our protocol (Fig 1) is a clinical classification rather than a cellularly specific diagnosis, but we believe it is more pathologically accurate than a classification made without MMM-based triangulation.

We recognize that this classification of mitochondrial dysfunction is most specific to the location of the microdialysis probe within the brain. It would likely be worth studying the intra-patient variations or lack thereof in identification of mitochondrial dysfunction between two different locations in the brain. It may also be beneficial to investigate how temporal patterns are associated with CMD derangements and NMS including MMM-identified mitochondrial dysfunction so that certain derangements may be anticipated according to time since injury and preemptively treated.

An important aspect of the present study has been our development of an algorithm for differentiating consistent from transient attributes (Fig 2). These attributes may be unimodal (e.g., LPR > 25) or multimodal, such as our definition for various NMS (Fig 1). Our algorithm is flexible and scalable in that its parameters can be modified in accordance with differing workflows or with the development of higher frequency or continuous CMD monitoring. Demarcating consistency can aid in artifact and noise reduction, improving the veracity of identified conditions to be treated. Conversely, transient spikes may themselves represent a distinct pathophysiological signal, perhaps indicating underlying volatility or moments where injury progression briefly overcomes treatment. The effect of applying the algorithm to our data was evident in that there was not only a significant difference in the prevalence of CMD derangements (Fig 3) but also in the prevalence of mitochondrial dysfunction (Table 6) before versus after filtering by consistency.

## Conclusions

We have compared the prevalence and multimodal context of several variations of mitochondrial dysfunction, differing in their classification criteria, across 592 patients from a superset of 619 TBI patients. Central to each criteria variant we have investigated is a derangement in the LPR, which is associated with worse outcome and is an indicator of cellular redox state. Importantly, our study has shown that a substantial proportion of TBI patients have a deranged LPR for the entirety of their monitoring, indicating a pressing need for neurometabolic therapies in intensive care. We have expanded on previous work by differentiating between consistent and transient derangements and by implementing the multimodal criteria of our neurometabolic classification protocol. In applying our stringent multimodal criteria to a cohort of 268 TBI patients, we found that almost half of all patients, or at least a fourth of patients if using modified CMD thresholds, demonstrate consistent mitochondrial dysfunction at some point during their care. Further research is called for into novel therapeutics and the prospective manipulation of multimodal parameters to manage brain metabolism and mitigate mitochondrial dysfunction in neurocritical care.
